# Sex Differences in Neuropsychiatric Symptoms Associated with Agitation in Frontotemporal Lobar Degeneration

**DOI:** 10.1002/alz70857_103036

**Published:** 2025-12-25

**Authors:** Celine N. Sakran, Juan‐Camilo Vargas‐González, Nico Paulo Dimal, Kasey Cortez, Hilary W. Heuer, Leah K. Forsberg, Brian S Appleby, Sami Barmada, Andrea Bozoki, David G. Clark, Yann Cobigo, Ryan Darby, Brad C. Dickerson, Kimiko Domoto‐Reilly, Douglas R. Galasko, Daniel H. Geschwind, Nupur Ghoshal, Neill R. Graff‐Radford, Ian M. Grant, David J Irwin, Ging‐Yuek Robin Hsiung, Lawrence S. Honig, Kejal Kantarci, Gabriel C Leger, Irene Litvan, Ian R MacKenzie, Joseph C. Masdeu, Mario F. Mendez, Chiadi U. Onyike, Belen Pascual, Peter S. Pressman, Eliana Marisa Ramos, Erik D Roberson, Emily J Rogalski, Brad F. Boeve, Adam L. Boxer, Howard J. Rosen, Carmela Tartaglia, ALLFTD Consortium Investigators

**Affiliations:** ^1^ Tanz Centre for Research in Neurodegenerative Disease, University of Toronto, Toronto, ON, Canada; ^2^ Toronto Western Hospital, University Health Network, Toronto, ON, Canada; ^3^ Memory Clinic, Toronto Western Hospital, University Health Network, Toronto, ON, Canada; ^4^ University Health Network, Toronto, ON, Canada; ^5^ Memory and Aging Center, Weill Institute for Neurosciences, University of California, San Francisco, San Francisco, CA, USA; ^6^ Department of Neurology, Mayo Clinic, Rochester, MN, USA; ^7^ Case Western Reserve University School of Medicine, Cleveland, OH, USA; ^8^ University of Michigan, Ann Arbor, MI, USA; ^9^ University of North Carolina, Chapel Hill, NC, USA; ^10^ Indiana Alzheimer's Disease Research Center, Indiana University School of Medicine, Indianapolis, IN, USA; ^11^ Weill Institute for Neurosciences, University of California, San Francisco (UCSF), San Francisco, CA, USA; ^12^ Vanderbilt University Medical Center, Nashville, TN, USA; ^13^ Massachusetts General Hospital and Harvard Medical School, Boston, MA, USA; ^14^ University of Washington, Seattle, WA, USA; ^15^ University of California, San Diego, La Jolla, CA, USA; ^16^ David Geffen School of Medicine, University of California, Los Angeles, Los Angeles, CA, USA; ^17^ Washington University School of Medicine, St. Louis, MO, USA; ^18^ Mayo Clinic in Florida, Jacksonville, FL, USA; ^19^ Mesulam Center for Cognitive Neurology and Alzheimer's Disease, Northwestern Feinberg School of Medicine, Chicago, IL, USA; ^20^ Penn FTD Center, Perelman School of Medicine, University of Pennsylvania, Philadelphia, PA, USA; ^21^ University of British Columbia, Vancouver, BC, Canada; ^22^ Columbia University Irving Medical Center, New York, NY, USA; ^23^ Department of Radiology, Mayo Clinic, Rochester, MN, USA; ^24^ University of California ‐ San Diego, La Jolla, CA, USA; ^25^ Houston Methodist Research Institute, Houston, TX, USA; ^26^ UCLA David Geffen School of Medicine, Los Angeles, CA, USA; ^27^ Johns Hopkins University School of Medicine, Baltimore, MD, USA; ^28^ University of Colorado School of Medicine, Aurora, CO, USA; ^29^ University of Alabama at Birmingham, Birmingham, AL, USA; ^30^ Healthy Aging & Alzheimer's Research Care (HAARC) Center, Healthy Aging & Alzheimer's Research Care (HAARC) Center, The University of Chicago, Chicago, IL, USA; ^31^ Memory and Aging Center, Department of Neurology, Weill Institute for Neurosciences, University of California, San Francisco, San Francisco, CA, USA; ^32^ Department of Neurology, Memory and Aging Center, University of California San Francisco, San Francisco, CA, USA; ^33^ Toronto Dementia Research Alliance, Toronto, ON, Canada; ^34^ Krembil Brain Institute, University Health Network, Toronto, ON, Canada; ^35^ Canadian Concussion Centre, Krembil Brain Institute, University Health Network, Toronto, ON, Canada

## Abstract

**Background:**

Agitation is a clinically significant symptom contributing to behavioral and psychological symptoms of dementia (BPSD) but is poorly understood across the different syndromes related to Frontotemporal Lobar Degeneration (FTLD). This study investigates sex differences in agitation across FTLD‐related syndromes and its relationship to various neuropsychiatric symptoms (NPS).

**Method:**

We analyzed data from 1,654 participants (916 males, 738 females; average ages 65.8 and 65.9, respectively) from the National Alzheimer's Coordinating Center (NACC) and ARTFL‐LEFFTDS Longitudinal Frontotemporal Lobar Degeneration (ALLFTD) study with a FTLD‐related syndrome: behavioral variant FTD (bvFTD), Primary Progressive Aphasia (non‐fluent variant (nfvPPA), semantic variant (svPPA)), Corticobasal syndrome (CBS) and Progressive Supranuclear Palsy (PSP). Participants’ symptoms were assessed using the Neuropsychiatric Inventory (NPI). Prevalence ratios and odds ratios were computed to assess the likelihood of NPS comorbidities when agitation was present in males and females. Principal Component Analysis (PCA) was performed to identify NPS associated with agitation and how they varied by sex.

**Result:**

Males were more likely to experience anxiety (bvFTD: *p* <0.001, CBS: *p* <0.01, PSP: *p* <0.0001), apathy (nfvPPA: *p* <0.01, PSP: *p* <0.01), depression (bvFTD: *p* <0.0001, PSP<0.01), disinhibition (nfvPPA: *p* <0.0001), PSP: *p* <0.001), and motor symptoms (bvFTD: *p* <0.01, CBS: *p* <0.01) when agitation was present. In contrast, females had a higher likelihood of experiencing disinhibition in svPPA (*p* <0.001). These findings suggest that agitation is associated with a wider range of NPS in males than in females. Agitation and NPS are especially prominent in males with PSP.

**Conclusion:**

This study reveals significant sex differences in NPS in FTLD‐related syndromes when agitation is present. Males and especially PSP, are more likely to experience a broader range of NPS in association with agitation. These findings underscore the need for further investigation into the underlying mechanisms driving these sex differences, particularly focusing on the neurobiological impact of agitation, the recognition of symptoms in the presence of greater behavioral disturbances, and potential variations from informant reports. Understanding these factors will provide valuable insights into the role of agitation in the presentation of NPS across FTLD‐related syndromes. Ultimately, addressing these gaps will enhance our ability to effectively treat and manage agitation in both male and female patients with FTLD‐related syndromes.

